# Convergence Between DSM-5 Section II and III Models of Personality Disorders Among Older Adults

**DOI:** 10.1093/geroni/igaa057.1258

**Published:** 2020-12-16

**Authors:** Lisa Stone, Daniel Segal

**Affiliations:** 1 University of Colorado Colorado Springs, Colorado Springs, Colorado, United States; 2 University of Colorado at Colorado Springs, Colorado Springs, Colorado, United States

## Abstract

Introduction: The Personality Inventory for DSM-5 (PID-5) is a measure of the alternative model of personality disorders (PDs), proposed in Section III of the DSM-5, but the PID-5 has limited evidence of validity for use among older adults. This study examined the validity of the alternate model through associations with the 10 traditional PDs in DSM-5. It was hypothesized that the PID-5 would relate to the traditional PDs in patterns predicted by the alternate model. Method: Older adults (N = 202) completed the PID-5 and the Coolidge Axis II Inventory (CATI), a measurement of the 10 PDs. Results: Correlations were computed between the PID-5’s 25 facets and the CATI’s 10 PD scales. All facets were found to significantly (p < .001) and positively correlate with all 10 PD scales, with large effect sizes (> .30). Next, regressions were conducted, with the PID-5 facets predicting each PD scale. Overall, across the 10 regression analyses, the PID-5 facets accounted for significant variance in the CATI PD scales, ranging from 64% (Avoidant) to 71% (Obsessive-Compulsive). Discussion: Although some DSM-5 hypothesized facets were significant predictors, many unexpected significant relationships were also detected. Of the 10 PD models, seven models included more unpredicted significant traits than predicted ones; two models included more significant predicted traits than unpredicted ones; one model included an equal number of predicted and unpredicted significant traits. We found substantially more overlap between the PID-5 and CATI than anticipated in unpredicted directions, suggesting that the PID-5 has good specificity but lacks sensitivity.

